# Hexyl gallate for the control of citrus canker caused by *Xanthomonas citri* subsp *citri*

**DOI:** 10.1002/mbo3.1104

**Published:** 2020-08-06

**Authors:** Lúcia B. Cavalca, Caio F. C. Zamuner, Luiz L. Saldanha, Carlos R. Polaquini, Luis O. Regasini, Franklin Behlau, Henrique Ferreira

**Affiliations:** ^1^ Departamento de Bioquímica e Microbiologia Instituto de Biociências Universidade Estadual Paulista Rio Claro Brazil; ^2^ Departamento de Química e Ciências Ambientais Instituto de Biociências, Letras e Ciências Exatas Universidade Estadual Paulista São José do Rio Preto Brazil; ^3^ Department of Research & Development Fundo de Defesa da Citricultura (Fundecitrus) Araraquara Brazil

**Keywords:** citrus protection, copper alternative, ester of gallic acid, sustainable agriculture

## Abstract

Brazil is the biggest producer of sweet oranges and the main exporter of concentrated orange juice in the world. Among the diseases that affect citriculture, Asiatic citrus canker, caused by the bacterial pathogen Xanthomonas citri, represents one of the most significant threats. The current Brazilian legislation regulating the control of citrus canker no longer requires the eradication of affected trees in states where the incidence of the disease is high. Instead, control involves disease control measures, including periodic preventative spraying of copper compounds. The long‐term use of copper for plant disease control has raised concerns about environmental accumulation and toxicity, as well as the selective pressure it exerts leading to the emergence of copper‐resistant X. citri strains. Here, we evaluated hexyl gallate (G6) as an alternative to copper compounds for citrus plant protection. G6 was able to protect citrus nursery trees against X. citri infection. Thirty days after inoculation, the trees treated with G6 developed 0.5 lesions/cm^2^ leaf area compared with the 2.84 lesions/cm^2^ observed in the untreated control trees. Also, G6 did not interfere with germination and root development of tomato, lettuce, and arugula, which is consistent with our previous data showing that G6 is safe for tissue culture cell lines. Membrane permeability tests showed that the primary target of G6 is the bacterial outer membrane. Finally, we could not isolate spontaneous X. citri mutants resistant to G6 nor induce resistance to G6 after long‐term exposures to increasing concentrations of the compound, which suggests that G6 may have multiple cellular targets. This study demonstrated that G6 is a promising candidate for the development and use in citrus canker management.

## Graphical abstract

We evaluated hexyl gallate (G6) as an alternative to copper compounds for citrus plant protection. G6 was able to protect citrus nursery trees against *Xanthomonas citri* infection, and it is safe for tissue culture cell lines. This study demonstrated that G6 is a promising candidate for the development and use in citrus canker management.

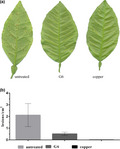

## 1 INTRODUCTION

Brazil is the leading producer of concentrated orange juice worldwide, supplying 80% of global demand. Among the threats affecting the Brazilian citrus industry, Asiatic citrus canker represents one of the most significant. The disease is caused by the Gram‐negative bacterium *Xanthomonas citri* subsp. *citri* (*X. citri*), which can infect all commercially important cultivars of citrus (Gottwald, Graham, & Schubert, 2002). This disease is fast becoming endemic in the main Brazilian citrus belt, a region comprising the states of São Paulo and west–southwest of Minas Gerais, which together generate more than 80% of Brazilian sweet orange production (Belasque & Behlau, 2011; Ference et al., 2018; Neves et al., 2010). According to current Brazilian legislation, states and areas where citrus canker is endemic are no longer obliged to eradicate canker‐affected or suspect trees (IN21, Ministry of Agriculture Livestock and Supply, MAPA; Brazil, 2018). Instead, growers have to adopt a set of control measures, which include frequent sprays of copper to minimize the impact of the disease on fruit production (Ference et al., 2018).

The effectiveness of copper to protect against *X. citri* infection is well‐demonstrated (Behlau, Amorim, et al., 2010; Behlau, 2010; Behlau, Scandelai, Da Silva, & Lanza, 2017; Graham, Dewdney, & Myers, 2010). However, after decades of heavy application of copper to protect crops around the world, including citrus, the emergence of copper resistance in plant pathogenic bacteria has become a matter of concern. Resistance to copper was first documented in *X. citri* about 20 years ago and has been noted in several studies since (Behlau, Canteros, Jones, & Graham, 2012; Behlau, Canteros, Minsavage, Jones, & Graham, 2011; Behlau, Jones, Myers, & Graham, 2012; Canteros, 1999). This fact is somewhat alarming as copper is the only pesticide recommended and used to control citrus canker in Brazil, and the accumulation of copper in the environment can result in toxic effects on microbial life, plants, animals, and other organisms (Clausen & Trapp, 2017; Rehman et al., 2019).

In light of emerging resistance and environmental toxicity, the development of sustainable alternatives to copper is now required. Our group has been investigating esters of gallic acid (alkyl gallates) as anti‐*X. citri* compounds for control of citrus canker (Savietto et al., 2018; Silva et al., 2013). Gallic acid (GA: 3,4,5‐trihydroxybenzoic acid) is a phenolic compound found in plants as a free acid or as a constituent of tannins (Kahkeshani et al., 2019). This natural product and its esters are commonly used in the food and pharmaceutical industries due to their well‐known antioxidant properties (Kahkeshani et al., 2019; Octyl‐gallate‐as‐food‐additive, 2015; Safety‐assessment‐of‐Propyl‐Gallate, 2007). For instance, esters of GA are in part responsible for the beneficial health effects associated with the green tea (Zhang, Zhang, Ho, & Huang, 2019). Moreover, GA and derivatives have been implicated as having anticarcinogenic, antimicrobial, antimutagenic, antiangiogenic, and anti‐inflammatory potential (reviewed by Ref. (Choubey, Varughese, Kumar, & Beniwal, 2015; Kahkeshani et al., 2019)). Following this, we have shown previously that alkyl gallates exhibit strong antibacterial activity against *X. citri* (Silva et al., 2013). Among the compounds we tested, hexyl gallate (G6) was selected for further study due to its prominent ability to inhibit infection by *X. citri* in plants exposed to G6 before experimental infection. Also, G6 was able to reduce bacterial load within citrus canker lesions when sprayed onto diseased leaves.

Here, we extended this evaluation of G6 as an antimicrobial alternative to copper compounds. We demonstrate in greenhouse trials that G6 sprays were able to protect citrus leaves from *X. citri* infection with comparable efficacy to that of copper. Phytotoxicity assays indicated that in contrast to copper, G6 does not interfere significantly with the development of indicative plants. Finally, membrane permeability analysis showed that the membrane of *X. citri* is the primary target of G6.

## 2 MATERIAL AND METHODS

### 2.1 Synthesis of hexyl gallate

**FIGURE 1:**
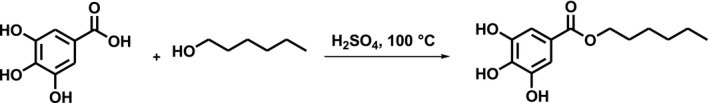
Synthesis of hexyl gallate (G6)

Hexyl gallate (G6) was synthesized by esterification of gallic acid with *n*‐hexanol (Figure 1) as previously described (Silva et al., 2013), with modifications. *n*‐Hexanol (90 mmol, 12 ml) was added to the gallic acid (30 mmol, 5.0 g). The suspension was stirred at 120°C for 15 min. Catalytic amounts of H_2_SO_4_ (3−5 drops) were added to the suspension and stirred at 100°C. The progress of the reaction was monitored by thin‐layer chromatography (TLC). After 5 days, the reaction medium was cooled to room temperature and 50 ml of ethyl acetate added. The residue was partitioned (5 × 50 ml) with 1 M sodium bicarbonate solution and then partitioned with deionized water (5 × 50 ml). The organic fraction was dried under reduced pressure, and this crude product was purified by column chromatography over silica gel using hexane and ethyl acetate (3:2). The structure of hexyl gallate was confirmed by NMR.

### 2.2 Bacterial strain and growth conditions

The *X. citri* strain used was the isolate 306 (IBSBF 1594) (Schaad et al., 2006), whose genome was sequenced by da Silva et al. (2002). The strain was propagated on NYG/NYG agar or in liquid medium (nitrogen–yeast–glycerol: 5 g/l of peptone, 3 g/l of yeast extract, 2% glycerol; for solid medium, bacterial agar was added to 15 g/l) at 29 °C. Liquid cultures were grown with shaking at 200 rpm for 14 hr.

### 2.3 Compound susceptibility assays

Bacterial growth inhibition by G6 was evaluated using the resazurin microtiter assay (REMA) as described in Silva et al. (2013), with minor modifications. Stock solutions of G6 were prepared in 100% ethanol (the concentrations of the stock solutions were adjusted to guarantee that after diluting them for testing, ethanol concentration would not exceed 1%). The concentration range of G6 varied from 100 to 0.78 μg/ml using a twofold scheme. The antibiotic kanamycin at a final concentration of 20 μg/ml and 1% ethanol were used as positive and negative controls, respectively. The resorufin relative concentration was determined based on fluorescence readings (excitation at 530 nm, emission at 590 nm) performed in a Synergy H1 Hybrid Multi‐Mode Microplate Reader (BioTek). The compound concentration/cell inhibition percentage ratio was described by logistical functions based on the average results of three independent experiments performed in triplicate. The inhibitory concentration (IC) was defined as the concentration of compound that completely inhibited bacterial growth/cell respiration in REMA. The IC values, as well as the percentages of growth inhibition, and the logistic dose–response curve were generated using the MS Excel. To assess the concentration of the compound that was lethal to *X. citri* (bactericidal dose), aliquots of the cell cultures were extracted from the REMA plates using a plate replicator and spread onto NYG agar before resazurin was added. Plates were incubated for up to 48 hr to increase the probability of rescuing *X. citri* after compound exposure.

### 2.4 Bacterial resistance

Natural resistance of *X. citri* against the compound was assessed by plating 100 μl of bacterial suspension (10^8^ CFU/ml) on NYG agar supplemented with G6 at the IC (100 μg/ml). Plates were incubated for 7 days at 29°C to score for the development of bacterial colonies. Kanamycin at 20 μg/ml was used as a positive control. Induction of resistance was performed according to Oz et al. (2014) with modifications. Experiments were initiated by exposing the bacteria to G6 in the range of 1/16–1/2 of the IC (6.25–50 μg/ml) using cultures of 100 μl at 10^6^ CFU/ml. Bacterial cultures were incubated for 48 hr at 29°C under constant double‐orbital rotation using a Synergy H1 Hybrid Multi‐Mode Microplate Reader (BioTek) (round 1). Next, the cultures were visually inspected to assess bacterial growth, and 40 µl of each bacterial suspension was spread onto NYG agar and incubated for up to 72 hr at 29 °C for CFU counting. Aliquots of 10 μl from the culture incubated in round one with the lowest G6 concentration were used as inocula to start four other 100 μl cultures, now containing twice the concentration of G6. This process was repeated for 31 days. Three independent experiments were performed.

### 2.5 Control of citrus canker

The efficiency of G6 at 600 µg/ml in 1% ethanol (10× the IC90 defined in this study) to protect against citrus canker was assessed using citrus nursery trees ("Pera" sweet orange) kept under greenhouse conditions. Copper at 0.7 g of metallic copper/l (Difere, 35% w/v metallic copper; Oxiquímica Agrociência LTDA, Jaboticabal) and 1% ethanol were used as positive and negative controls, respectively. Each treatment was applied by spraying 100 young susceptible leaves at one‐half expansion size (20 per nursery tree) until the run‐off point. After 24 hr, leaves were spray‐inoculated with an *X. citri* suspension at 10^8^ CFU/ml in 0.85% NaCl. Disease severity was assessed 30 days after inoculation by determining the number of canker lesions per cm^2^ leaf area using the software ImageJ (https://imagej.nih.gov/ij/index.html). The average severity of citrus canker for each treatment was calculated using all assessed leaves. The standard error of the means was calculated for all treatments using GraphPad Prism 6.

### 2.6 Membrane integrity analysis

Cells of *X. citri* were cultivated until the OD_600 nm_ 0.4, then diluted 10X in fresh NYG medium, and exposed to G6 for 15 and 30 min. Membrane integrity was assessed using the LIVE/DEAD BacLight Bacterial Viability Kit following the manufacturer's instructions (L7007, Molecular Probes). Cells were immobilized in agarose‐covered slides and observed using an Olympus BX‐61 microscope equipped with a monochromatic camera Orca‐Flash 2.8 (Hamamatsu). Images were acquired and processed using the software CellSens Dimension Version 11 (Olympus).

### 2.7 Phytotoxicity

Phytotoxicity was evaluated on three model plants, Arugula (*Eruca sativa*), cherry tomato (*Solanum lycopersicum*), and lettuce cultivar Grand Rapids (*Lactuca sativa*). Commercial seeds (Isla Pak) were placed in Petri dishes on the surface of filter paper previously wetted with sterilized tap water. Treatments were applied by adding 1 ml of G6 (600 µg/ml in 1% ethanol; 10X the IC90), copper at 0.7 g of metallic copper/l, and 1% ethanol (vehicle control) to the filter paper disks. Petri dishes were kept in the greenhouse to allow for the development of roots. After the germination of seeds, roots were measured, and data were analyzed using ANOVA with Dunnett's multiple comparison test using the software GraphPad Prism version 6. Experiments were performed in triplicate and repeated three times.

## 3 RESULTS

### 3.1 G6 has bactericidal activity against *X. citri*

**FIGURE 2:**
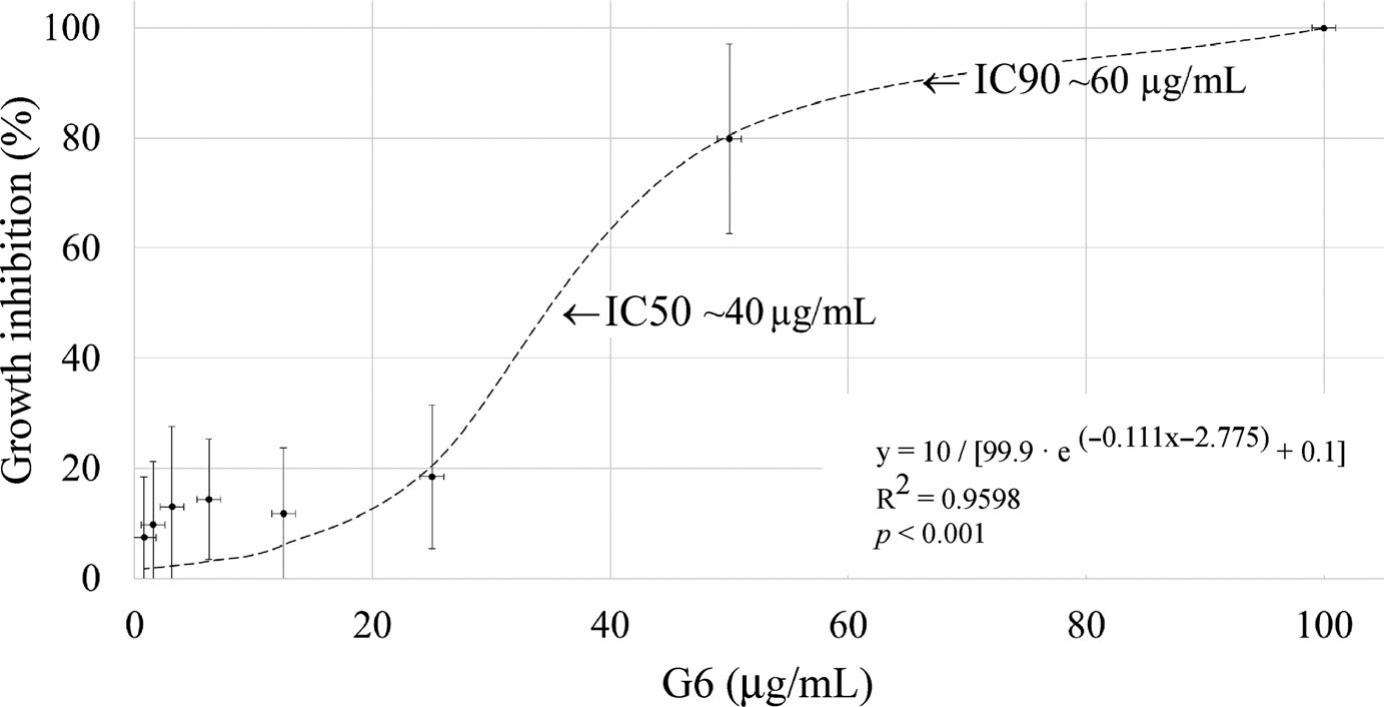
Growth inhibition profile of G6 against *Xanthomonas citri*. Cells were exposed to various concentrations of G6 (100, 50, 25, 12.5, 6.25, 3.12, 1.56, and 0.78 μg/ml) for a period of 12 hr, and growth inhibition was monitored by REMA. Dots represent the mean percentages of growth inhibition, and the vertical lines, the standard deviation of the calculated mean based on three independent experiments. Dashed line, a logistic model used to fit the G6 dose–response data distribution. IC90 and IC50, the concentrations of G6 that promote 90% and 50% bacterial growth inhibition, respectively

To define the minimal inhibitory concentration (MIC) of G6, *X. citri* was exposed to different concentrations of the compound in REMA analysis (Figure 2). G6 inhibited *X. citri* growth in a dose–response manner, showing a more pronounced activity at the range of 30–50 μg/ml. The non‐linear logistic function *y* = 10/[99.9 · *e* ^−0.111^ *^x^* ^−2.775^ + 0.1] had the best fit to the G6 dose–response data, which allowed the calculation of the IC, IC90, and IC50 values at approximately 100, 60, and 40 μg/ml, respectively. These concentrations were used as references for the pathogenicity, phytotoxicity, resistance, and microscope tests described below. Of note, the IC values of G6 against *X. citri* were the same order of magnitude as the positive control kanamycin (20 μg/ml). Finally, we found 1% ethanol, used to dissolve G6, did not affect *X. citri* growth.

To determine the minimal bactericidal/bacteriostatic concentration (MBC) of G6, aliquots of the *X. citri* cultures used in REMA were transferred to the NYG agar medium just before resazurin was added to measure bacterial respiration. After 48 hr of incubation, cellular regrowth was observed for all the samples in which cells were exposed to G6 at <100 μg/ml (bacteriostatic effect). Conversely, no live cells could be rescued after treatment with the highest dose assayed in REMA (100 μg/ml), which was then considered a bactericidal rate. This concentration coincided with the IC determined above.

### 3.2 G6 protected citrus leaves against citrus canker

**FIGURE 3:**
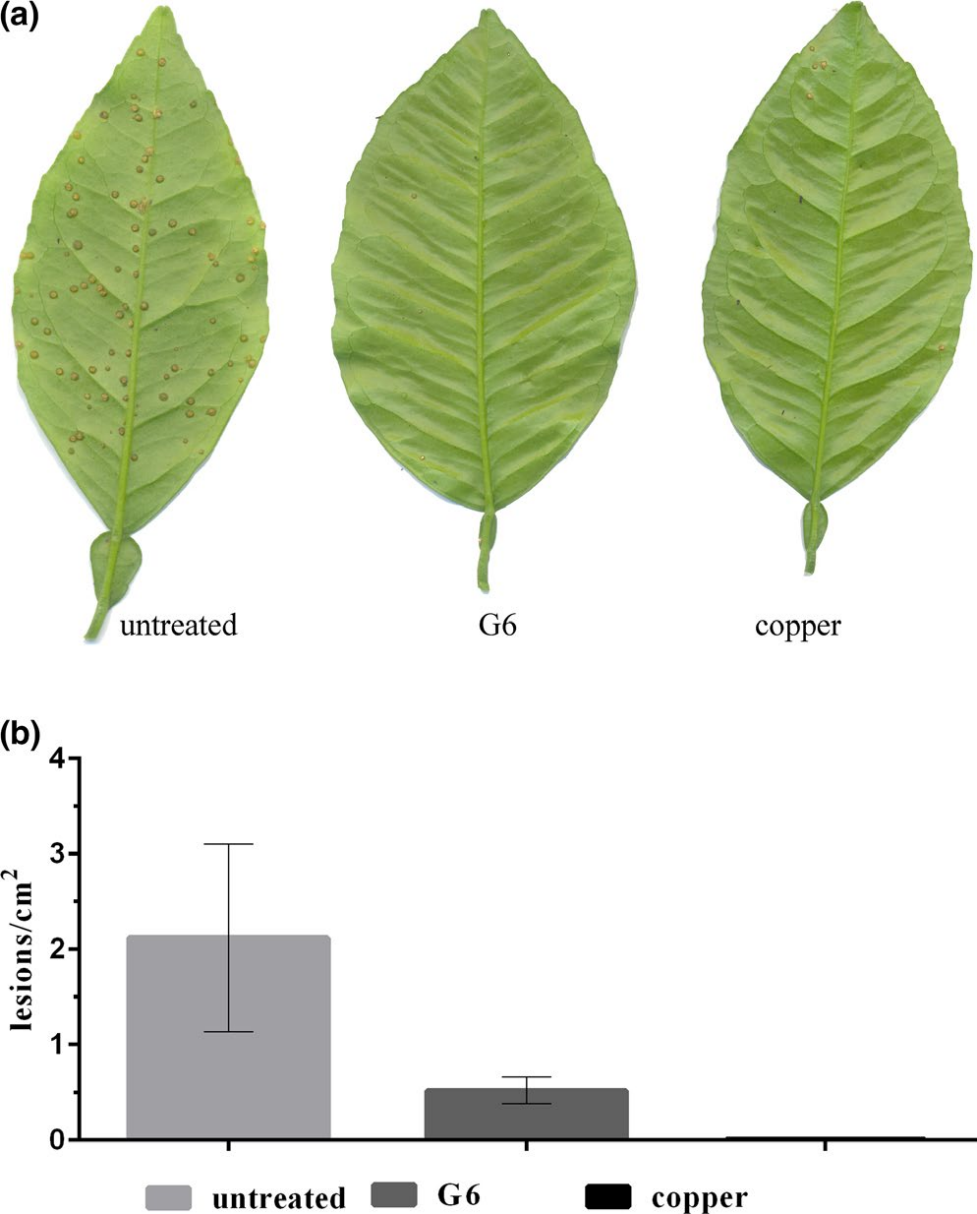
Hexyl gallate (G6) protects citrus plants against *Xanthomonas citri* infection. Nursery trees of *Citrus sinensis* cv. Pera were sprayed with G6 (600 µg/ml) and the copper formulation Difere until the run‐off point. Twenty‐four hours after treatment, plants were spray‐inoculated with *X. citri* (10^8^ CFU/ml) and kept under greenhouse conditions for 30 days to score for disease symptom development. (a) A representative experiment in which leaves were detached from an untreated plant (sprayed with 1% ethanol; vehicle control), and from plants protected with G6 and copper. (b) Citrus canker lesions per cm^2^ of leaf area. Bars: the average of lesions/cm^2^; vertical lines, the standard error of the averages

We previously showed that *X. citri* pre‐exposed to a sublethal dose of G6 (IC80) and infiltrated into sweet orange leaves, a susceptible host, were not able to induce citrus canker symptoms (Silva et al., 2013). Following that, in this study, we observed that G6 was able to protect citrus nursery trees against *X. citri* infection. At 30 days after inoculation, trees treated with G6 developed 0.5 lesions/cm^2^ of leaf area as opposed to 2.84 lesions/cm^2^ observed in trees treated with the vehicle control. Trees sprayed with copper had 0.005 lesions/cm^2^ (Figure 3).

### 3.3 Hexyl gallate is not phytotoxic

**FIGURE 4:**
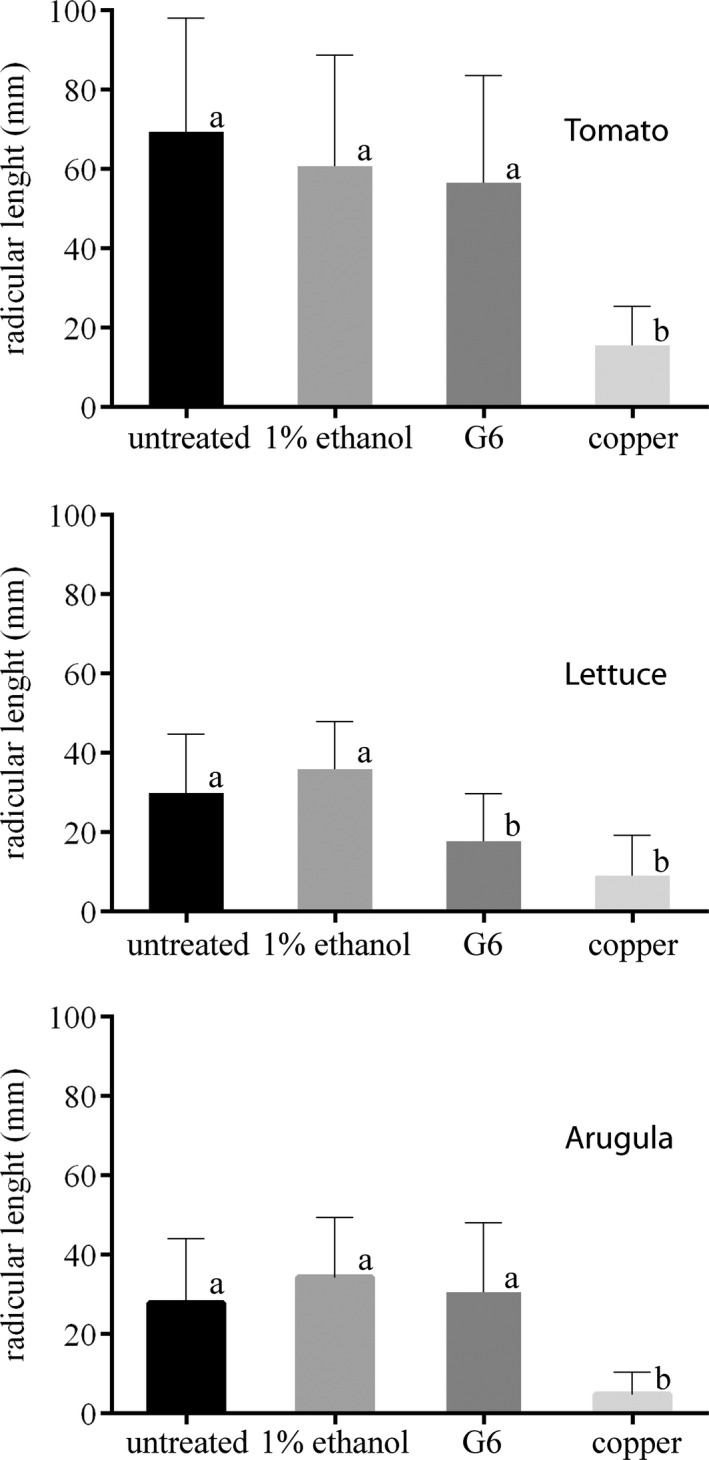
Phytotoxicity of G6. Effect of G6 upon the radicular development of tomato, lettuce, and arugula seeds. Whiskers indicate the standard deviation of the mean. Untreated, sterilized tap water; 1% ethanol (vehicle control); G6 (600 µg/ml); and copper. Different letters indicate significant statistical difference (*p* < 0.05) by ANOVA with Dunnett's multiple comparison test (n = 50)

The phytotoxic potential of G6 and copper was assessed by seed germination and root growth tests, using tomato, lettuce, and arugula as targets. The germination of seeds exposed to G6 was similar to untreated or negative controls (1% ethanol) for all test plants (data not shown). Gallate did not affect root development of tomato and arugula, but it seemed to slightly retard the growth of lettuce roots. Conversely, copper promoted a significant reduction in the average root length of all plant species tested (Figure 4).

### 3.4 Hexyl gallate targets the membrane of *X. citri*

**FIGURE 5:**
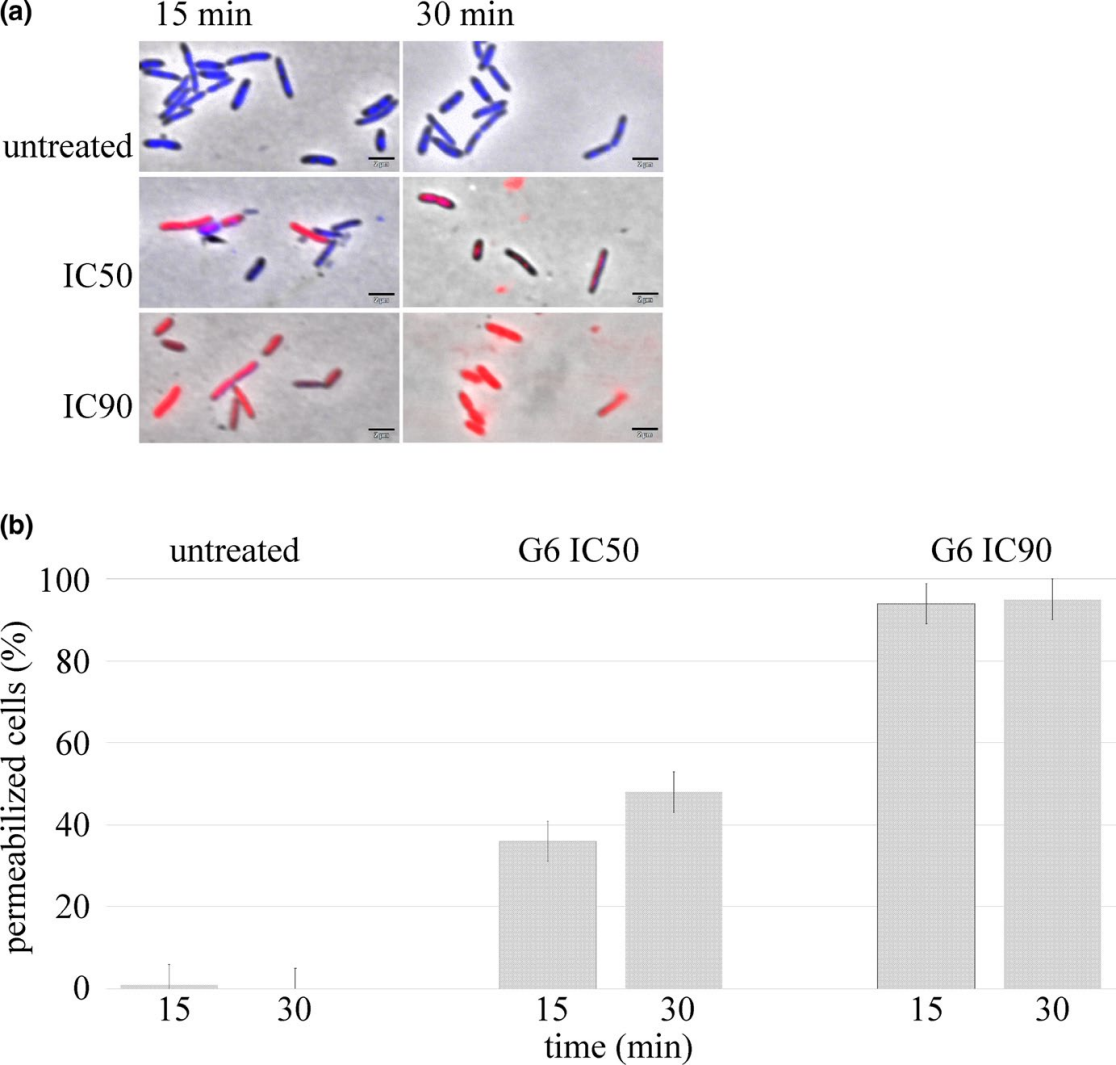
G6 permeabilizes the membrane of *Xanthomonas citri*. (a) Cells were exposed to 1% ethanol (untreated; vehicle control), and G6 at 40 and 60 μg/ml (IC50 and IC90, respectively) for 15 and 30 min. After treatment, cells were stained with the nucleoid dyes SYTO9 and IP before microscope observation. Cells artificially colored in red are permeable to IP, while cells colored in blue show only SYTO9 labeling and membrane integrity. Scale bar, 2 μm; magnification 100×. (b) Bars represent the percentage of cells with a disrupted membrane, and the whiskers indicate the standard error of the mean (at least 200 cells were analyzed per treatment)

We have previously shown that G6 targets the cell division process of *X. citri* (Silva et al., 2013). It has been demonstrated that G6 also permeabilizes the membrane of the Gram‐positive bacterial model *Bacillus subtilis* (Krol, 2015). To investigate whether G6 had a similar dual effect on *X. citri*, cells were exposed to sublethal doses of the compound and evaluated for membrane permeability to propidium iodide (IP), a nucleic acid dye that enters the cells only when the membrane is disrupted (Figure 5). *X. citri* cells exposed to negative controls had normal morphology with a standard average cell size of ~1.5 μm, and practically no cells with permeabilized membranes (Figure 5a,b). On the contrary, G6 was able to permeabilize the membrane of *X. citri* after 15 min of exposure (Figure 5a, IC50, and IC90). Membrane permeabilization to IP was time‐ and concentration‐dependent. Increasing the dose of G6 from IC50 to IC90 or exposing the cells for a longer period with a particular dose of G6 led to a significant increase in the proportion of permeabilized cells (Figure 5B).

### 3.5 Natural resistance and resistance induction to G6

Because G6 is intended for use in citrus canker control, we wanted to assess the likelihood of *X. citri* developing resistance to this compound. The exposure of *X. citri* at increasing concentrations of G6 was carried out continuously for 31 days. No growth was observed when the concentration of G6 approached the IC (100 μg/ml). This indicates that the compound does not induce resistance in *X. citri*. Additionally, to assess whether naturally resistant cells could be isolated, a suspension of *X. citri* was directly plated on NYG agar supplemented with G6 at the IC. Likewise, no *X. citri* colonies were observed after incubation of the plates for 7 days.

## 4 DISCUSSION

The current Brazilian legislation that regulates the control of citrus canker in the country determined for the first time in 60 years that growers in states where the incidence of the disease is high are no longer required to identify and remove affected trees. Instead, growers have to adopt a set of control measures to reduce the impact of the disease on fruit quality and yield (Ference et al., 2018). Among these, repeated spraying of copper is the most commonly used measure in states within Brazil but also in Argentina, Uruguay, and Florida (USA), where citrus canker is endemic. Therefore, there is increasing concern regarding this regular use of copper in orchards. Besides the development of resistant strains, as previously mentioned, frequent applications, commonly associated with overestimated rates and spray volumes, may lead to toxicity to roots due to the accumulation of copper in the soil over time (Alva, 1993; Lamichhane et al., 2018). Toxic amounts of copper impair the development of roots and the canopy as a result of the reduction in chlorophyll, the content of water in the cells, and uptake of nitrogen, iron, and other important nutrients (Hippler et al., 2018). High copper levels in the soil may also jeopardize carbon mineralization and soil fertility by affecting the respiration of soil‐borne microorganisms (Dumestre, Sauve, McBride, Baveye, & Berthelin, 1999). It is also worth mentioning that at excess concentrations in plant tissues, copper increases the production of reactive oxygen species, which intensifies plant stress and canopy development (Hippler et al., 2018).

As an alternative to copper, we evaluated the potential of an ester of gallic acid, hexyl gallate (G6), to protect citrus plants against *X. citri* infection. We demonstrated that G6 impeded *X. citri* entry into the leaves of a susceptible citrus host and reduced the severity of citrus canker by nearly six times in comparison with untreated controls. Although copper performed slightly better, G6 treatments have several significant advantages. Gallates of short carbon side chains, such as G6, are considered to be fairly safe, displaying antimicrobial action against several pathogenic microorganisms, as well as antitumoral activity (Aldulaimi et al., 2017; Chew, Mahadi, Wong, & Goh, 2018; Denardi‐Souza, Luz, Manes, Badiale‐Furlong, & Meca, 2018; Kim, Par, Choi, Kwak, & Kim, 2019; Peredo‐Silva et al., 2017; Silva et al., 2013). Gallates are also used as food supplements and cosmetic ingredients due to their antioxidant activity (Octyl‐gallate‐as‐food‐additive, 2015; Safety‐assessment‐of‐Propyl‐Gallate, 2007). Furthermore, our group demonstrated that G6 is non‐genotoxic, non‐mutagenic, and a pro‐apoptotic agent, which exhibits chemopreventive action that could also help in chemotherapy (Silva, Polaquini, Regasini, Ferreira, & Pavan, 2017).

The mechanism of action of G6 was explored further in the present work. Previously, we showed that gallates of short carbon side chains, including G6, were able to perturb the divisome of *X. citri* (Silva et al., 2013). Next, we showed that G6 could act similarly disrupting cell division in the Gram‐positive bacterial model *B*.* subtilis* (Krol et al., 2015). Although G6 could interact with the cell division protein FtsZ from *B*.* subtilis*, as demonstrated using fluorescence shifts following compound/protein mixture, G6 also induced membrane permeabilization in this species. In line with this, data from other groups also pointed out that alkyl gallates of short carbon side chains target the membranes of Gram‐positive bacteria (Kubo, Fujita, Nihei, & Nihei, 2004; Takai, Hirano, & Shiraki, 2011). Here, we demonstrated that G6 can also permeabilize the membrane of the Gram‐negative pathogen *X. citri*. Curiously, the acetylation of G6 in another study produced a compound that had the bacterial membrane as a target, and it lost the ability to interact with FtsZ in *X. citri* (Savietto et al., 2018). Altogether, data indicate that the primary target of G6 in *X. citri* is the bacterial membrane, even though it may interact with the division protein FtsZ.

Besides being an environmentally friendly compound, derived from gallic acid, our results indicate that G6 is a multitarget active ingredient. This implies that there is no or very low risk for the development of G6 resistance in *X. citri* populations in the field over time as demonstrated in this study. The resistance of plant bacteria to copper and antibiotics, the main pesticides used to manage bacterial diseases, has been widely documented (Behlau et al., 2011; Behlau, Hong, Jones, & Graham, 2013; Burr, Norelli, Katz, Wilcox, & Hoying, 1988; Cha & Cooksey, 1991; Schroth, Thomson, & Moller, 1979; Stall & Thayer, 1962) and is an important concern. Another relevant aspect of G6 relies on the fact that both targets in the bacterial cell, the membrane, and the divisome are highly conserved among plant pathogenic bacteria, which indicates a potential of G6 to be used for the control of several other bacterial diseases affecting crops, contributing to the urgent need of replacement of copper and antibiotics in agriculture (Lamichhane et al., 2018).

In conclusion, this study demonstrated that G6 is a promising candidate for integration into citrus canker management programs. More importantly, because G6 is a non‐pollutant, multitarget compound, it may contribute to the sustainability of crop protection worldwide with the potential to be used against several bacterial diseases affecting crops without impacting negatively on the environment in contrast to copper and antibiotics. Further studies are underway to develop a suitable formulation of G6 that can be used for field studies where its efficiency needs to be ultimately assessed.

## CONFLICT OF INTEREST

None declared.

## AUTHOR CONTRIBUTIONS

**Lúcia B. Cavalca:** Conceptualization (equal); formal analysis (lead); investigation (lead); methodology (equal); validation (equal); writing‐original draft (equal). **Caio F. C. Zamuner:** Investigation (equal); methodology (equal). **Luiz L. Saldanha:** Investigation (equal); methodology (equal). **Carlos R. Polaquini:** Investigation (equal); methodology (equal). **Luis O. Regasini:** Investigation (equal); methodology (equal). **Franklin Behlau:** Formal analysis (equal); validation (equal); writing‐original draft (equal); writing‐review & editing (lead). **Henrique Ferreira:** Conceptualization (lead); formal analysis (equal); funding acquisition (lead); project administration (lead); supervision (lead); writing‐original draft (equal); writing‐review & editing (lead).

## ETHICS STATEMENT

None required.

## Data Availability

All data generated or analyzed during this study are included in full in this published article.
